# Highly Sensitive Detection of Melanin in Melanomas Using Multi-harmonic Low Frequency EPR

**DOI:** 10.1007/s11307-024-01911-3

**Published:** 2024-03-22

**Authors:** Mohammad Wehbi, Lionel Mignion, Nicolas Joudiou, Evelyne Harkemanne, Bernard Gallez

**Affiliations:** 1 Louvain Drug Research Institute, Biomedical Magnetic Resonance Research Group, Université Catholique de Louvain (UCLouvain), Brussels, Belgium; 2 Nuclear and Electron Spin Technologies (NEST) Platform, Louvain Drug Research Institute, Université Catholique de Louvain (UCLouvain), Brussels, Belgium; 3 Dermatology Department, Cliniques Universitaires Saint-Luc, Brussels, Belgium

**Keywords:** EPR, ESR, *In vivo*, Melanoma, Clinical EPR, Melanin, Dermatology, Multi-harmonic

## Abstract

**Purpose:**

Low frequency EPR can noninvasively detect endogenous free radical melanin in melanocytic skin lesions and could potentially discriminate between benign atypical nevi and malignant melanoma lesions. We recently succeeded in demonstrating the ability of clinical EPR to noninvasively detect the endogenous melanin free radical in skin lesions of patients. However, the signal-to-noise ratio (SNR) was extremely low warranting further research to boost the sensitivity of detection. In the present study, we assessed the performance of a clinical EPR system with the capability to perform multi-harmonic (MH) analysis for the detection of melanin.

**Procedures:**

The sensitivity of MH-EPR was compared with a classical continuous wave (CW)-EPR (1st harmonic) detection in vitro in melanin phantoms, *in vivo* in melanoma models with cells implanted in the skin, in lymph nodes and having colonized the lungs, and finally on phantoms placed at the surface of human skin.

**Results:**

*In vitro*, we observed an increase in SNR by a factor of 10 in flat melanin phantoms when using MH analysis compared to CW combined with an increase in modulation amplitude. In B16 melanomas having grown in the skin of hairless mice, we observed a boost in sensitivity *in vivo* similar to that observed *in vitro* with the capability to detect melanoma cells at an earlier stage of development. MH-EPR was also able to detect non-invasively the melanin signal coming from melanoma cells present in lymph nodes as well as in lungs. We also observed a boost of sensitivity using phantoms of melanin placed at the surface of human skin.

**Conclusions:**

Overall, our results are paving the way for new clinical trials that will use MH clinical EPR for the characterization of pigmented skin lesions.

**Supplementary Information:**

The online version contains supplementary material available at 10.1007/s11307-024-01911-3.

## Introduction

Melanoma incidence is continuously increasing over time with a worldwide total of 325.000 new cases in 2020 [1, 2]. Skin melanomas detected at an early stage of development are easily cured by surgical resection with an excellent survival prognosis (10-year survival rate of almost 98%) [3]. However, advanced melanomas (with lymph node invasion and distant metastases) show limited survival rates [4]. Over the past decade, immunotherapy and targeted therapies have significantly improved the prognosis of metastatic patients (5-year survival rate of 43 to 64%) [5, 6]. Unfortunately, these therapies are not always curative, very expensive and affect the patient’s quality of life [7]. Therefore, early diagnosis remains essential to increase the chance to cure melanoma patients. In clinical practice, in addition to visual inspection, dermoscopy is the most widely non-invasive technique used by dermatologists to inspect skin lesions [8, 9]. When a lesion is suspect of being a melanoma, the lesion is excised with minimal margins. The histopathology will confirm if the lesion is benign or malignant. In addition, histological analysis will inform on the tumor thickness (known as Breslow depth) which is a main prognostic factor for skin melanoma staging [10]. When the Breslow depth is smaller than 0.8 mm, a second surgery is performed to enlarge the margins of resection. When the Breslow thickness is greater than 0.8 mm, the second surgery with larger margins is often associated with a sentinel lymph node biopsy as it represents the first site of metastases in patients with melanoma.

In this context, advanced technologies are under development to increase the accuracy of the diagnostic, to better characterize the lesions, to visualize their invasiveness in the epidermis and to early assess lymph node colonization by melanoma cells. Among the innovative methods, clinical low frequency Electron Paramagnetic Resonance (EPR) that characterizes the melanin content in the lesion, has the potential to be an adjunct diagnostic method of melanoma [11, 12]. Melanin is composed of a mixture of polymers derived from the oxidation of L-tyrosine by the enzyme tyrosinase [13]. The melanogenic process leads to two different forms: brown/black eumelanin and yellow/reddish pheomelanin that both contain stabilized semi-quinone free radicals that present different EPR spectra [14]. The single EPR line (with g = 2.005) recorded from human pigmented melanoma has been ascribed to eumelanin [14]. Pigmented melanomas are the most frequently diagnosed lesions, the incidence of amelanotic melanomas being very low (less than 2%) [15]. The noninvasive detection of melanin by EPR could potentially help in the characterization of pigmented lesions in two ways. First, it was shown that the EPR signal recorded *in vitro* (9 GHz) on paraffin-embedded human melanoma biopsies was significantly lower in benign nevi than in melanomas [16]. Moreover, in these biopsies, the EPR signal was significantly different between low Breslow and high Breslow depth melanomas [16]. Second, as the thickness of the skin melanoma is the most important prognostic factor in patient management, there is a major interest for a method that could map the distribution of melanoma cells in tissues. This could be indirectly be achieved through EPR imaging of melanin in pigmented lesions. In this context, several studies performed at 9 GHz on paraffin-embedded melanomas or freeze-dried samples have demonstrated the capability of EPR imaging to provide images of pigmented melanomas [17–19]. EPR images from melanoma samples remarkably overlaid over the histological sections [19]. It has been a major challenge to translate these results obtained *in vitro* on biopsies by high frequency EPR into living subjects using low frequency EPR systems that are compatible with a noninvasive detection of the EPR signal. This was achieved in L-Band (1 GHz) operating spectrometers equipped with surface coil resonators placed at the surface of melanoma lesions implanted at the surface of skin of mice [17, 20]. Finally, recently, the first clinical study has been carried out on patients presenting suspect lesions of melanomas (n = 100) using a clinical EPR system (1 GHz) operating in a traditional continuous-wave mode [11]. This study demonstrated for the first time the ability of this technology to detect noninvasively an endogenous free radical (i.e. melanin) in human subjects by EPR. The EPR signal of melanin was significantly higher in melanoma lesions compared to benign atypical nevi [11]. A trend toward a higher signal intensity (though not significant) was observed in high Breslow depth melanomas than in low Breslow lesions [11]. This proof-of-concept study also revealed that the signal-to-noise ratio (SNR) obtained from melanin in lesions was very low warranting further research to achieve a better sensitivity of detection.

The use of multi-harmonic analysis could potentially increase the SNR recorded in EPR [21–26]. In most classical EPR spectrometers, a first harmonic signal is obtained as a result of a phase sensitive detector operating at the magnetic field modulation frequency [27]. Combining information from multiple harmonics of the field-modulated signal has been proposed as a method to obtain the first derivative spectrum with minimal distortion and improved SNR [22]. The harmonics are obtained by digital phase-sensitive detection of the signal at the modulation frequency and its integer multiples. Since each harmonic of the EPR signal is affected by the same level of noise, by measuring multiple harmonics and averaging the results, the impact of the noise is reduced. This leads to a higher SNR because the signal components remain consistent across the different harmonics while the noise, being random, averages out [21–26]. In addition, while the use of a modulation amplitude larger than the signal linewidth causes a broadening of the EPR linewidth and a distortion of the signal [27], multi-harmonic analysis allows the use of large modulation amplitude without spectral distortion which highly improves the sensitivity of the acquired signal [24]. Overall, multi-harmonic analysis can also highlight weaker signals that might be overshadowed in conventional EPR measurements and/or subtract background signals or artifacts more effectively. In this context, we have recently upgraded our clinical EPR system with the capability of performing multi-harmonic analysis. The objectives of the present study were to compare the performances of multi-harmonic analysis with classical CW on the clinical EPR system for melanin detection. The studies were performed *in vitro* and *in vivo* in systems of increasing complexities in conditions similar to those used in clinical exams to be performed on patients (within a minimum time for tuning and for recording the EPR spectrum). The analysis was performed *in vitro* on flat melanin phantoms mimicking melanoma lesions, on melanoma models inoculated in the skin of mice, on lymph nodes invaded by melanoma cells (mimicking the first step of metastatic migration), on lungs invaded by metastatic melanoma cells, and finally using flat melanin phantoms placed at the surface of human skin.

## Material and Methods

### Melanin Phantoms

Phantoms were prepared as solid dilutions of eumelanin (Sepia officinalis, > 99%; Sigma-Aldrich, Steinheim, Germany; 3.5 × 10^18^ spins/gram of melanin) by trituration with increasing amounts of sucrose (> 99%, Fluka Biochemika, Steinheim, Germany). The powder was weighed on Scotch® Magic tape with melanin quantity ranging between 75 µg to 1 mg. The diameter of the circle occupied by the powder was chosen as less than 8 mm (diameter of the loop coil). The scotch tape used did not present any EPR signal. After weighing, the tape was covered with a second tape and the obtained film was used for measurement purposes.

### Cell Culture

Two melanoma cell lines were used: B16F10luc pigmented melanoma cells and nonpigmented WM-266–4 melanoma cells were purchased from ATCC (Manassas, Virginia, USA) and Rockland Immunochemicals (Limerick, PA, USA) respectively. B16F10Luc and WM2664 melanoma cell lines were grown in Dulbecco’s Modified Eagle Medium (DMEM; Gibco™, Thermo Fisher Scientific, Waltham, MA, USA) supplemented with 10% fetal bovine serum (FBS; Gibco™, Thermo Fisher Scientific, Waltham, MA, USA). Cells were incubated at 37 °C with 5% CO_2_. Cells were used for inoculation in animals after 3 passages of cell culture.

### Tumor Inoculation in Mice

All animal experiments were carried out in accordance with the local ethics committee for animal care of the Université catholique de Louvain (Protocol #2023/UCL/MD/06) and with the European Directive 2010/63/EU.

#### Intra Dermal Skin Injection

Mice were anesthetized with inhaled isoflurane mixed with air (3% for induction, 1.5% for maintenance). Five-week-old female NMRI nude mice (n = 8) (Elevage Janvier, Le Genest St. Isle, France) were inoculated via an intradermal injection of 200 k cells in 50 µL PBS of murine luciferase-encoding B16F10Luc pigmented melanoma cells or 200 k in 50 µL PBS cells of human WM2664 melanoma cells (used as non-pigmented control cells). Tumors were measured as 2 perpendicular diameters (length and width) of the tumor, to determine the surface area in mm^2^.

#### Subcutaneous Lymph Node Injection

For the proof-of-principle, we used an injection of cells into the lymph nodes to insure the exact localization of melanoma cells. Mice were anesthetized with inhaled isoflurane mixed with air (3% for induction, 1.5% for maintenance). Five-week-old female NMRI nude mice (n = 8) (Elevage Janvier, Le Genest St. Isle, France) were injected at the tail base with 20 µL of 0.5% Evans Blue dye (Alfa Aesar, Haverhill, MA, USA). After 24 h, a small incision was performed in the iliac region. The lymph nodes were identified as small blue dots because of the accumulation of the dye. 200 k cells in 20 µL PBS of murine luciferase-encoding B16F10Luc melanoma cells were injected directly in one of the right or the left iliac lymph node. After injection, the wound was closed with a sterile suture thread.

#### Lung Metastasis Model

Mice were anesthetized with inhaled isoflurane mixed with air (3% for induction, 1.5% for maintenance). Five-week-old female NMRI nude mice (n = 8) (Elevage Janvier, Le Genest St. Isle, France) were injected intravenously with 750 k cells in 50 µL PBS of Luciferase-encoding murine B16F10Luc melanoma cells in the tail vein. Melanoma cells are known to accumulate in the lungs after IV injection [28].

#### Measurement on Human Skin

Films containing melanin described previously were taped onto the surface of a human arm belonging to a healthy volunteer (Phototype III) who signed an informed consent form.

### Multi-harmonic and CW EPR Measurements *in vivo *and *in vitro*

**Fig. 1:**
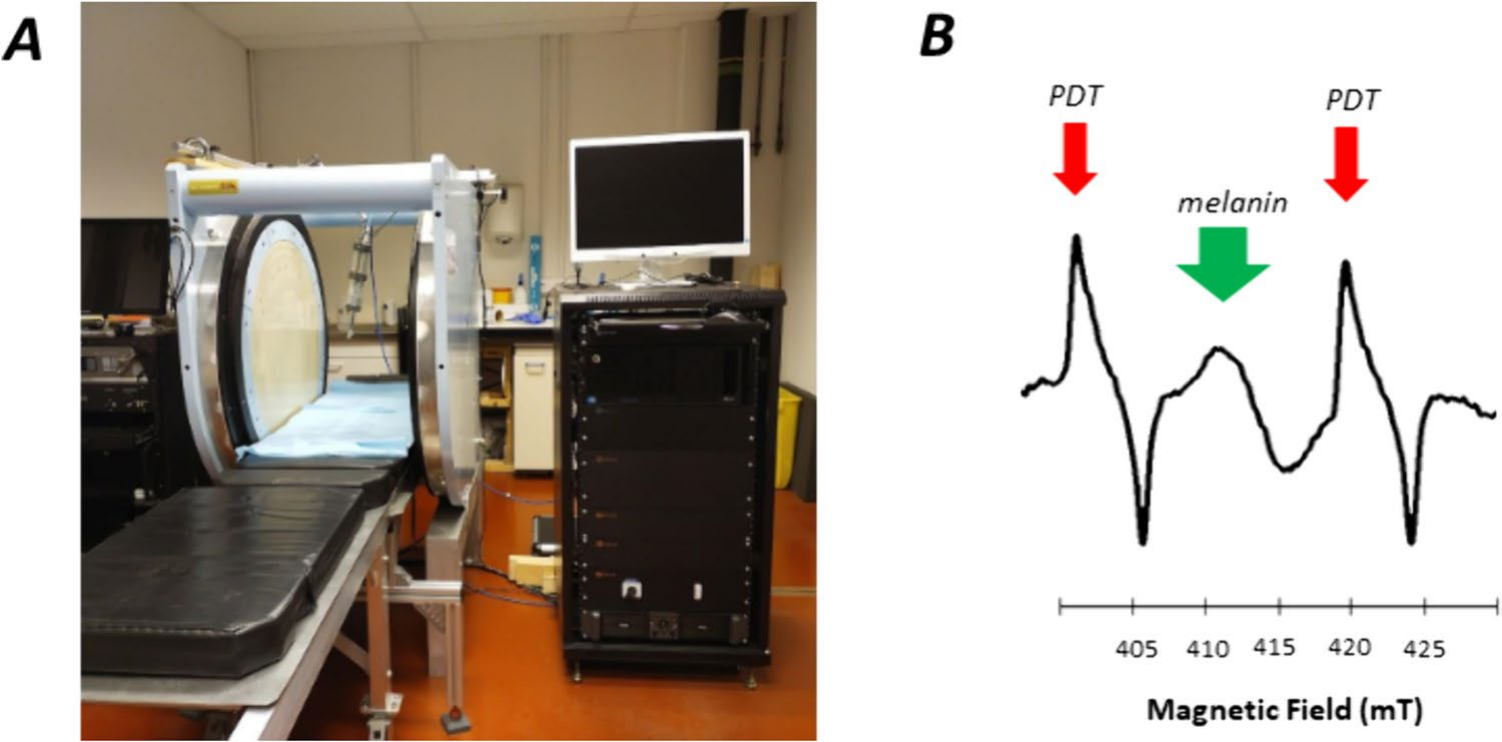
**A**: Clinical EPR spectrometer (Clin-EPR, on the left) equipped with a module for multi-harmonic analysis (Novilet, on the right). **B**: EPR spectrum recorded *in vivo* on skin melanoma in mice. The EPR spectrum contains signals coming from melanin (center, green arrow) and the doublet of ^15^N-PDT (low and high field signals, red arrows; external reference placed in a sealed capillary attached to the coil)

EPR measurements were performed using a clinical EPR spectrometer (Clin-EPR LLC, Lyme, NH) operating at 1.15 GHz equipped with the eSpect^++^ expansion module for multi-harmonic EPR analysis (Novilet, Poznan, Poland) (Fig. 1a). Measurements were carried out using the following parameters: center field, 41.6 mT; modulation amplitude, 0.3 mT or 0.6 mT; microwave power, 10 mW; modulation frequency, 20.4 kHz; time constant, 5 ms; scan time, 5 s; sweep width, 5 mT; number of acquisitions, 5 scans. Automatic tuning was performed by the system, followed by a manual adjustment for optimization. A sealed capillary containing 10 μL of a 2 mM solution of ^15^N-PDT (4-oxo-2,2,6,6-tetramethylpiperidine-d_16_-^15^N-1-oxyl; CDN Isotopes, Pointe-Claire, Canada) was fixed along the axis of the resonator with the tip of the capillary just at the surface of the loop coil. ^15^N-PDT was used for field positioning knowing that the EPR signal of melanin appears between the two lines of PDT (Fig. 1b) [11]. For *in vitro* calibrations, films containing melanin were placed on top of a solid surface and ten independent EPR measurements were performed by acquisitioning a series of five scans. For *in vivo* measurements, the area of interest (skin tumor or area identified thanks to the bioluminescence for the lymph node and metastatic models) was placed under a loop-gap surface coil resonator (Novilet, Poznan, Poland) with an inner diameter of 8 mm placed in the center of the magnet. A film (Parafilm® “M,” Neenah, WI, USA) 10 × 20 cm was folded three times resulting in eight layers, and placed between the coil and the area of interest. This method was previously optimized to allow rapid tuning of the coil on lossy biological samples [4, 13]. Mice were anesthetized with inhaled isoflurane mixed with air (3% for induction, 1.5% for maintenance). Measurements were recorded at days 5, 8, 11, 13, 15 after B16F10Luc tumor induction in the skin, 5, 8, 11, 14 days after WM2664 tumor induction in the skin, and 5, 7, 9, 11, 14 days after B16F10Luc tumor injection in the lymph nodes. The tumor diameter was measured with a digital caliper (Traceable® Products, 12,554 Galveston Road Suite B230 Webster, TX 77598 USA). Two acquisition modes were used: 1st harmonic mode (CW) and multi-harmonic mode, both possible on the same resonator. First, the 1st harmonic signal was measured with a modulation amplitude of 0.3 mT (corresponding to the experimental conditions used in the previous clinical trial carried out on patients with lesions suspects of being melanomas) [11]. As the clinical EPR system was upgraded with the capability to deliver larger modulation amplitude, another 1st harmonic analysis was applied at modulation amplitude of 0.6 mT. For multi-harmonic analysis, the analysis range for multi-harmonic data processing was set up to 100 harmonics, and the cut off frequency of the low-pass filter used in the post-acquisition was 700 kHz. Peak-to-peak signal amplitude of the recorded signal was measured and calculated as a ratio over the noise recorded.

### Bioluminescence Measurements

While superficial melanoma can be easily visualized, the invasion in deeper tissues such as in lymph nodes and lungs required to use bioluminescence to assess melanoma cells expansion. That is the reason why we selected a B16 melanoma model expressing luciferase (B16F10luc). Tumors developed in the lymph node and metastasis models were monitored by bioluminescence imaging, using IVIS Spectrum *in vivo* Imaging System (PerkinElmer, Waltham, MA, USA). The mice were anesthetized with inhaled isoflurane mixed with air (3% for induction, 1.5% for maintenance). D-luciferin (PerkinElmer, 150 mg/kg) was injected intra-peritoneally (IP). After 25 min, the mice were placed in the bioluminescence imaging setup, and the bioluminescence signal was measured as the total flux of photons detected from the tumor (p/s) (the tumor area is specified in a defined region of interest on the camera image). A preliminary kinetics experiment allowed us to the optimal time point for *in vivo* imaging.

## Results

### *In vitro*, the SNR of Melanin was Increased by a Factor of 10 Using Multi-harmonic EPR Compared to CW-EPR

**Fig. 2:**
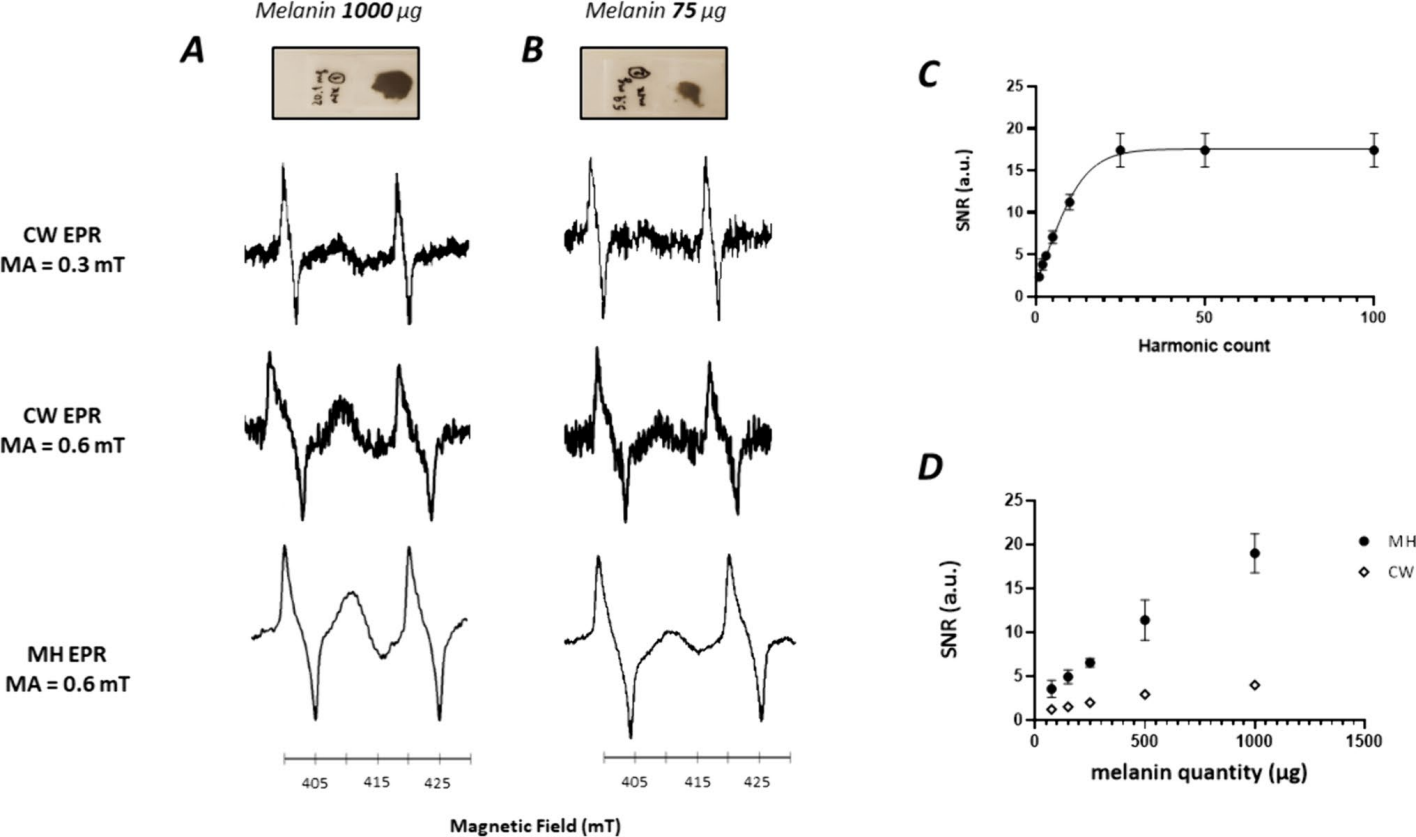
EPR studies on melanin phantoms. **A**: Studies on phantom of melanin (1 mg). From top to bottom: sample, EPR spectrum recorded in CW mode (0.3 mT modulation amplitude), EPR spectrum recorded in CW mode (0.6 mT modulation amplitude), EPR spectrum reconstructed with 100 harmonics (0.6 mT modulation amplitude). **B**: Studies on phantom of melanin (75 µg). From top to bottom: sample, EPR spectrum recorded in CW mode (0.3 mT modulation amplitude), EPR spectrum recorded in CW mode (0.6 mT modulation amplitude), EPR spectrum reconstructed with 100 harmonics (0.6 mT modulation amplitude). **C**: Influence of the number of harmonics used on the SNR of melanin phantom (1 mg) (mean of 3 measurements ± SD). **D**: Evolution of SNR as a function of melanin quantity in the phantom. Closed symbols: MH analysis (0.6 mT modulation amplitude). Open symbols: CW analysis (0.3 mT modulation amplitude). Mean of 3 measurements ± SD

Melanin phantoms of varying content (0.075 mg, 0.15 mg, 0.25 mg, 0.5 mg, 1 mg) were placed under the resonator and their EPR signal was measured. As our clinical EPR system has been upgraded with the capability to deliver higher modulation amplitude (0.6 mT) than previously used (0.3 mT) in the clinical trial on pigmented lesions [11]), we recorded the signal using a modulation amplitude of 0.3 mT, 0.6 mT using CW mode or using multi-harmonic detection. Typical EPR spectra recorded on phantoms containing 75 µg and 1 mg of melanin are shown in Fig. 2A-B. The relationship between the SNR recorded and the number of harmonics used for the reconstruction is shown in Fig. 2C for the phantom containing 1 mg of melanin. The SNR dramatically increased up to the 25th harmonic and then reached a plateau. We further used 100 harmonics for each reconstruction. A clear increase in SNR was observed using multi-harmonic analysis compared to CW mode. For example, while the EPR signal recorded on phantoms of 75 µg was at the limit of the noise, an obvious EPR signal was observed using multi-harmonic analysis. Of note, as we used a modulation amplitude of 0.6 mT because we focused on the melanin signal, the signal of melanin was not distorted contrarily to the PDT signal. Using a smaller modulation amplitude for reconstruction, the signal of PDT was not distorted (Fig. S1). A strong linear relationship between the SNR and the melanin quantity was observed in both multi-harmonic mode (Pearson R^2^ = 0.93, p < 0.0001) and CW mode (Pearson R^2^ = 0.91, p < 0.0001) (Fig. 2D). By comparing both slopes of the calibration curves, we found that the SNR has increased by a factor of 10 using the multi-harmonic mode compared to CW using a modulation amplitude of 0.3 mT (Fig. 2D). The combined use of higher modulation amplitude (factor 2) and detection of 25 harmonics (that theoretically could lead to an increase in SNR by a factor 5) led to this increase in SNR by a factor 10.

### The Sensitivity of Detection of Melanin in Skin Melanoma is Largely Increased Using Multi-harmonic EPR

**Fig. 3:**
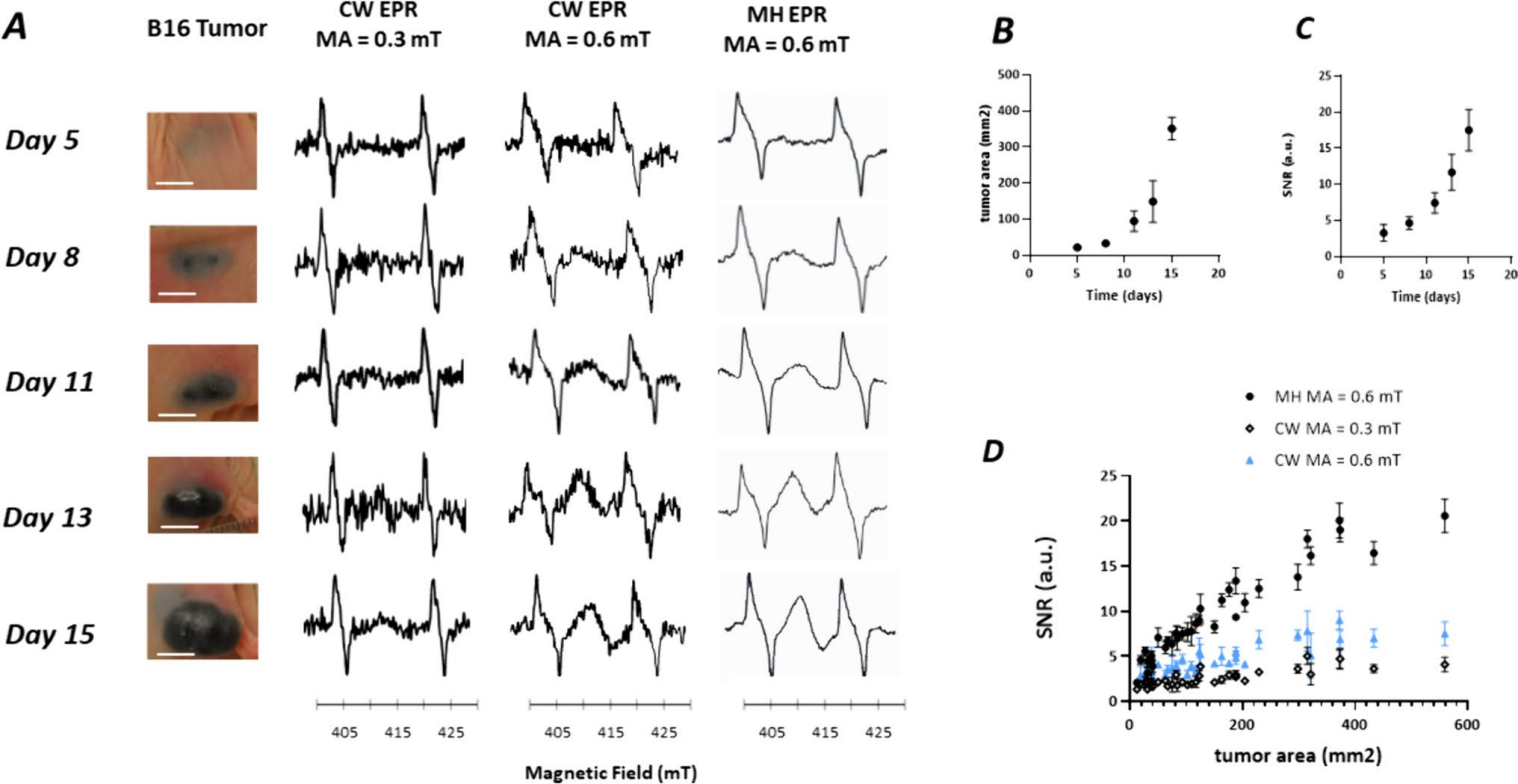
*In vivo* studies on pigmented B16F10 melanomas in the skin of mice (n = 8). **A**: From left to right: illustrative pictures of the skin melanoma over time (days after inoculation, scale bar = 5 mm), EPR spectrum recorded in CW mode (0.3 mT modulation amplitude), EPR spectrum recorded in CW mode (0.6 mT modulation amplitude), EPR spectrum reconstructed with 100 harmonics (0.6 mT modulation amplitude). **B**: evolution of tumor area over time. **C**: evolution of SNR of the melanin signal over time (MH analysis). **D**: Relationship between the SNR of melanin and the tumor area. Each point represents individual tumor. Closed symbols: MH analysis (0.6 mT modulation amplitude). Open symbols: CW analysis (0.3 mT modulation amplitude). Blue symbols: CW analysis (0.6 mT modulation amplitude)

**Fig. 4:**
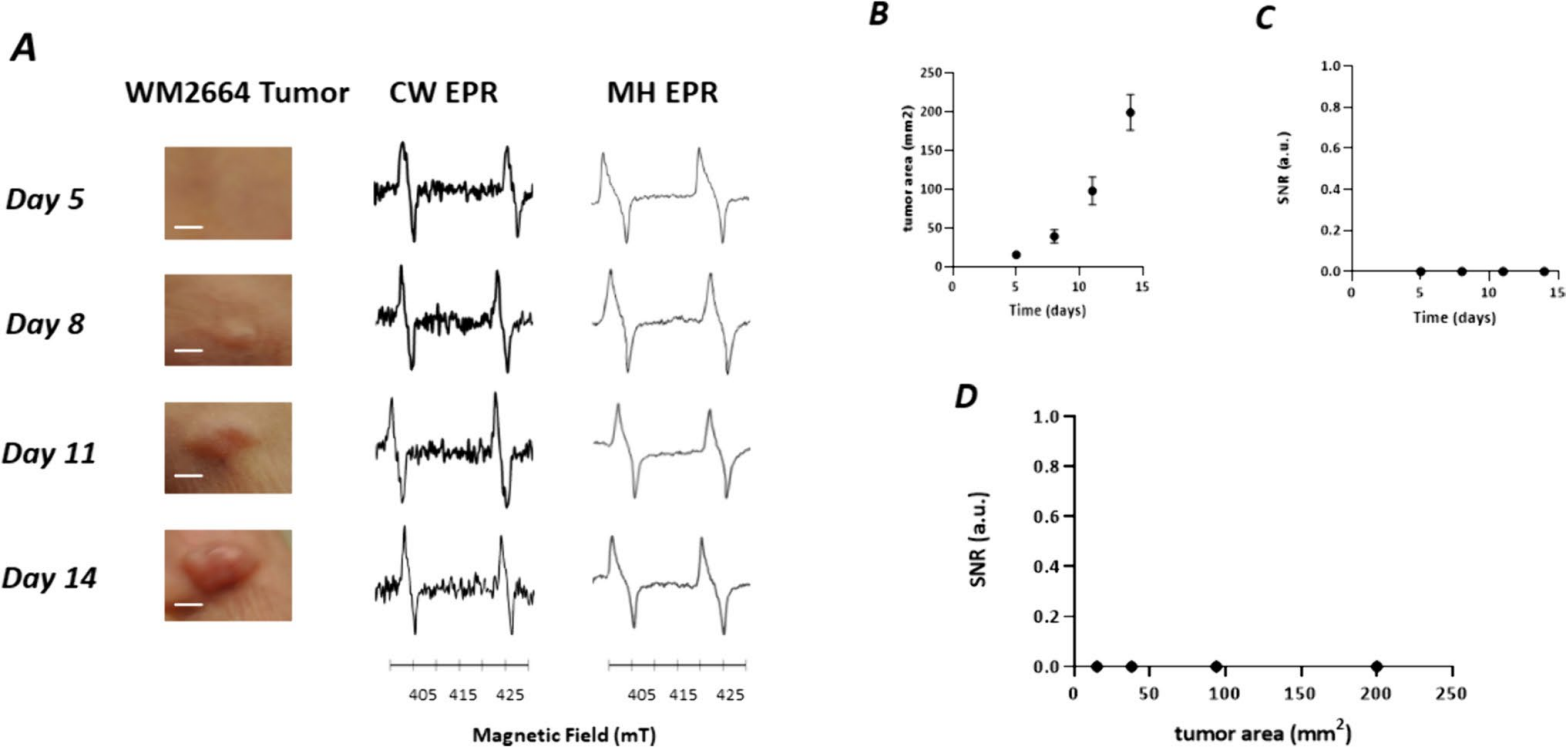
*In vivo* studies on non-pigmented WM2664 melanomas in the skin of mice (n = 8). **A**: From left to right: illustrative picture of the skin melanoma over time (days after inoculation, scale bar = 5 mm), EPR spectrum recorded in CW mode (0.3 mT modulation amplitude), EPR spectrum recorded in CW mode (0.6 mT modulation amplitude), EPR spectrum reconstructed with 100 harmonics (0.6 mT modulation amplitude). **B**: evolution of tumor area over time. **C**: evolution over time of SNR recorded in the region of melanin of the EPR spectrum (MH analysis). The SNR remained zero for this non-pigmented melanoma. **D**: Relationship between the SNR of melanin and the tumor area. Each point represents individual tumor using MH analysis (0.6 mT modulation amplitude)

We recorded the EPR signal using both multi-harmonic and CW mode in skin melanomas during the tumor growth. This was achieved using B16F10 pigmented tumors (n = 8), and WM2664 non-pigmented tumors (n = 8) used as controls. Illustrative results obtained on the pigmented B16F10 melanomas are presented in Fig. 3A. The EPR signal of melanin was clearly visible 8 days after inoculation of tumors cells in the skin using multi-harmonic analysis while the signal was confounded with the noise using CW at the same stage of development. As expected, the EPR signal of melanin increased over time with same trend than the tumor growth (Fig. 3C-C). In Fig. 3D, we plotted the SNR recorded as a function of the tumor area for all tumors analyzed during their tumor growth. The EPR signal in both modes increased along with the increase of tumor surface area measured on the mice skin (Pearson r = 0.94, p < 0.0001, for multi-harmonic and Pearson r = 0.73, p < 0.0001 for CW). As shown in Fig. 3D, the SNR recorded *in vivo* in skin melanoma was much higher using MH analysis than the SNR recorded using CW mode (modulation amplitude = 0.3 mT or 0.6 mT). To confirm that the EPR signal recorded was indeed corresponding to melanin, we also used the non-pigmented WM266-4 model as a control. The results obtained for the non-pigmented model are presented in Fig. 4, using the same presentation of the results used for the pigmented melanoma in Fig. 3. No EPR signal was recorded from melanin on the skin surface of the mice during the tumor growth, in both multi-harmonic and CW mode.

### Multi-harmonic EPR Detected Melanoma Cells in Lymph Nodes Earlier than CW-EPR

**Fig. 5:**
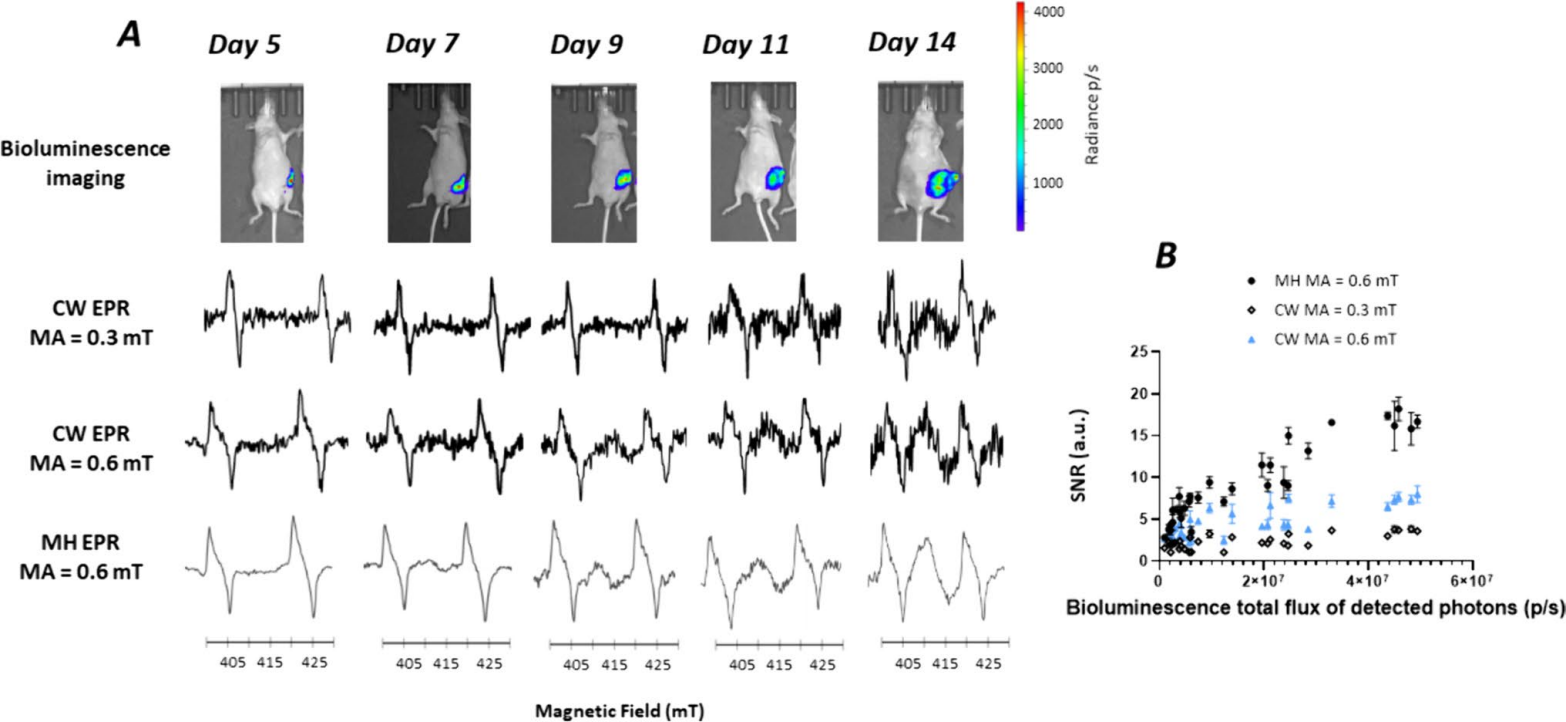
*In vivo* studies on lymph nodes inoculated with B16F10 melanoma cells in mice (n = 8). **A**: From top to bottom: illustrative bioluminescence images showing the evolution of invasion of the lymph node over time, EPR spectrum recorded in CW mode (0.3 mT modulation amplitude), EPR spectrum recorded in CW mode (0.6 mT modulation amplitude), EPR spectrum reconstructed with 100 harmonics (0.6 mT modulation amplitude). **B**: Relationship between the SNR of melanin and the flux of photons. Each point represents individual tumor. Closed symbols: MH analysis (0.6 mT modulation amplitude). Open symbols: CW analysis (0.3 mT modulation amplitude). Blue symbols: CW analysis (0.6 mT modulation amplitude). Mean of 3 measurements ± SD

Contrarily to superficial skin melanomas, melanoma cells growing in lymph nodes are not visible. To monitor the growth of melanoma cells in the iliac lymph nodes, we used bioluminescence as we selected a B16F10 melanoma expressing luciferase. After each bioluminescence measurement, the EPR signal was monitored by placing the surface coil over the iliac region of mice (n = 8). It was possible to obtain a visible EPR signal starting from day 7 after inoculation using the multi-harmonic analysis. The SNR recorded by the multi-harmonic mode was always greater than that of the CW mode as shown in Fig. 5A. The EPR signal in both modes increased along with the increase of bioluminescence total flux of the photons detected from the tumor, with a higher correlation coefficient for the multi-harmonic analysis (Pearson r = 0.91, p < 0.0001) compared to CW with a modulation amplitude of 0.3 mT (Pearson r = 0.7, p < 0.0001) (Fig. 5B). The SNR recorded was also higher using MH analysis than the SNR recorded using CW using a modulation amplitude of 0.6 mT (Fig. 5B).

### Multi-harmonic EPR Allowed the Detection of Melanoma Cells in Lungs

**Fig. 6:**
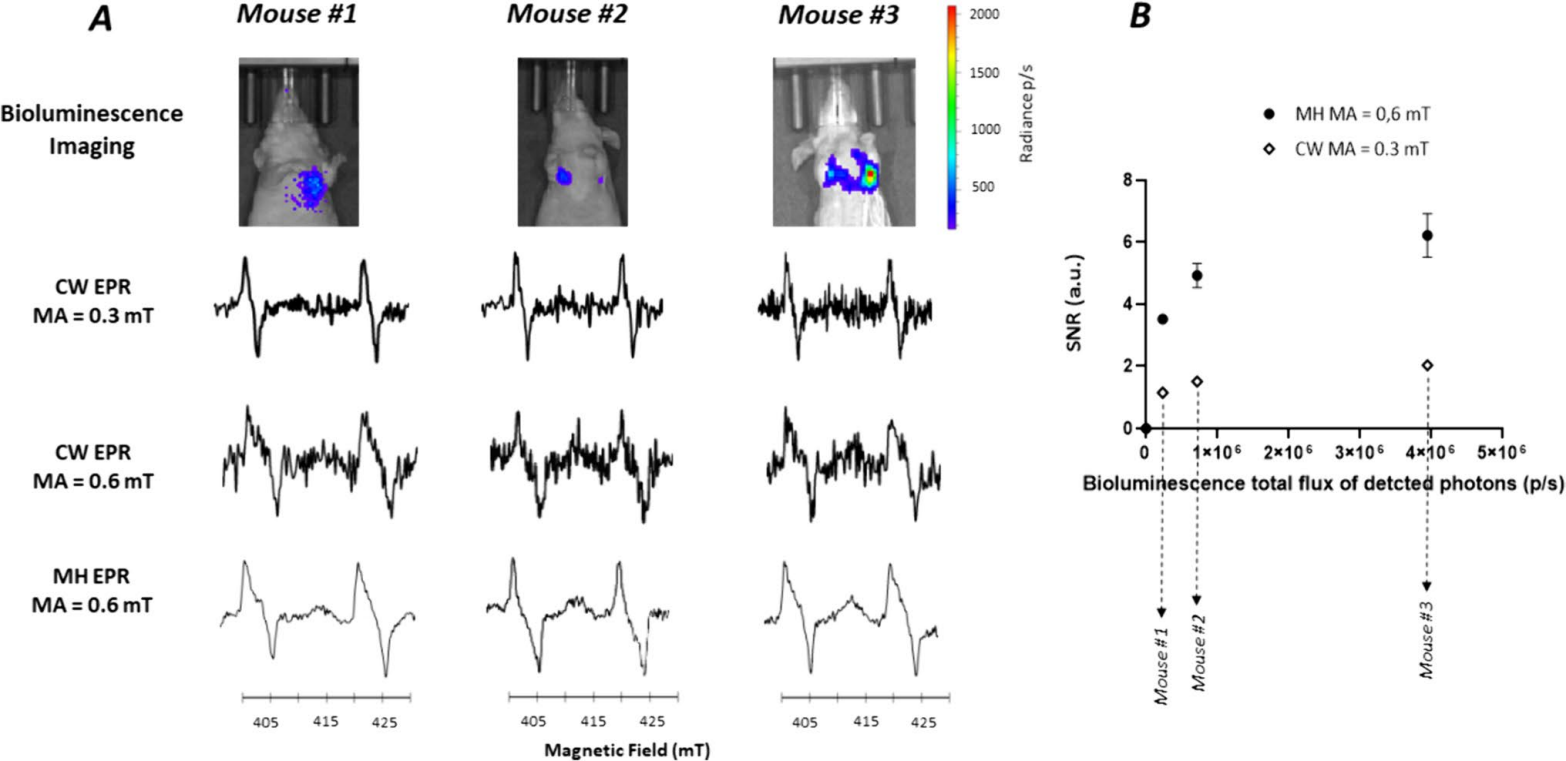
*In vivo* studies on lungs invaded by melanoma cells. **A**: From top to bottom: illustrative bioluminescence images showing the invasion of the lungs, EPR spectrum recorded in CW mode (0.3 mT modulation amplitude), EPR spectrum recorded in CW mode (0.6 mT modulation amplitude), EPR spectrum reconstructed with 100 harmonics (0.6 mT modulation amplitude). **B**: Relationship between the SNR of melanin and the flux of photons. Each point represents individual tumor. Closed symbols: MH analysis (0.6 mT modulation amplitude). Open symbols: CW analysis (0.3 mT modulation amplitude). Mean of 3 measurements ± SD

Having succeeded in getting an EPR signal from melanoma cells having invaded lymph nodes, we sought to a possible detection in deeper tissues using a metastatic model of melanoma in the lungs. B16F10luc were injected intravenously in the tail vein. The bioluminescence signal coming from the lung was measured during the proliferation of the melanoma cells. The EPR measurements were difficult to obtain because of the breathing motion in this region of the animals. Over 8 mice injected, we succeeded in getting both bioluminescence and EPR spectra only in 3 animals (37.5%). The results are presented in Fig. 6 with a comparison of using CW with a modulation amplitude of 0.3 or 0.6 mT, or using multi-harmonic analysis. For these mice, the SNR was between 1 and 2 using CW analysis while it was possible to record an EPR spectrum with higher SNR using multi-harmonic analysis (Fig. 6A). In Fig. 6B, we present the results correlating the bioluminescence total flux of the detected photons from the tumor cells and the SNR recorded using CW and multi-harmonic analysis.

### Multi-harmonic EPR Allows the Sensitive Detection of Melanin in Phantoms Placed at the Surface of Human Skin

**Fig. 7:**
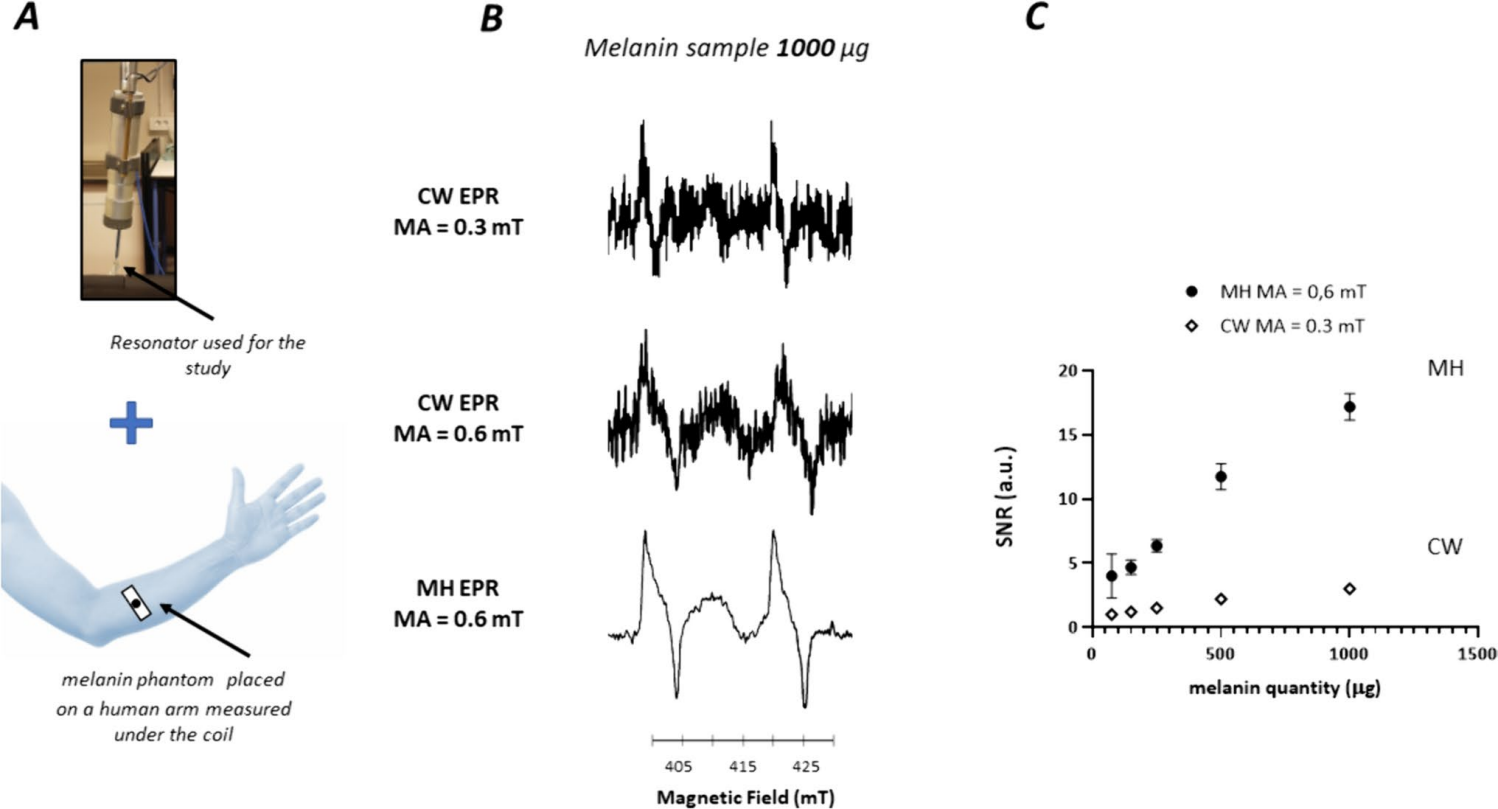
**A**: Design of the studies performed on phantoms of melanin taped at the surface of a human arm. Top: resonator used for the study. Bottom: the coil was placed over a melanin phantom (tape) placed at the surface of of the skin. **B**: EPR spectrum recorded in CW mode (0.3 mT modulation amplitude), EPR spectrum recorded in CW mode (0.6 mT modulation amplitude), EPR spectrum reconstructed with 100 harmonics (0.6 mT modulation amplitude). **C**: Relationship between the SNR of melanin and content of melanin in the phantom. Closed symbols: MH analysis (0.6 mT modulation amplitude). Open symbols: CW analysis (0.3 mT modulation amplitude)

As our ambition is to perform a new clinical trial on pigmented lesions, we carried out a simple assay mimicking experimental conditions used on patients by placing phantoms of melanin at the surface of the skin of a human arm (Fig. 7 A). Melanin phantoms with varying content (0.075 mg, 0.15 mg, 0.25 mg, 0.5 mg, 1 mg) were positioned under the surface coil resonator. As expected from the previously described experiments, we obtained a higher SNR using multi-harmonic analysis compared to CW-EPR (Fig. 7B). A linear relationship between SNR of melanin signal and melanin quantity of phantoms present *in vivo* was maintained clearly in both multi harmonic mode (Pearson R^2^ = 0.95, p < 0.0001) and CW mode (Pearson R^2^ = 0.97, p < 0.0001) (Fig. 7C). Also, the SNR of the melanin signal of the 1000 mg phantom using the multi harmonic analysis was significantly higher than that of the CW mode (Fig. 7 B) (unpaired t test, p < 0.0001).

## Discussion

The main result of the present study is that clinical EPR can significantly benefit from using a multi-harmonic analysis to boost the sensitivity of detection. Most developments in clinical EPR have been related so far to the field of oximetry [29–31] and retrospective dosimetry [32, 33]. Another potential application of clinical EPR that we are developing since several years is the ability to characterize skin melanomas through the assessment of melanin [11, 12, 17–20]. Detecting noninvasively the endogenous free radical melanin is a real challenge. While we succeeded in its noninvasive detection in skin melanoma in a previous clinical trial (Eudra-CT2017-002503–94) [11], the SNR was extremely low. In order to boost the sensitivity of detection, the clinical EPR system installed in Brussels has been upgraded with the capability to use larger modulation amplitude and to perform multi-harmonic analysis using the eSpect^++^ module from Novilet (Poznan, Poland). Using a higher number of harmonics provided a dramatic increase in SNR (Fig. 2C). Besides the advantages previously described in the introduction, the use of multi-harmonic analysis could in principle offer the possibility to use higher power and a modulation amplitude larger than the EPR linewidth with the capability to recover an EPR signal without distortion [21, 22]. The modulation amplitude we used (0.6 mT) in the present study was twice the one previously used in the clinical trial on pigmented skin lesions. It should be emphasized the gain remains limited considering that the intrinsic EPR linewidth of eumelanin is about 0.5–0.6 mT. This means that the signal of melanin obtained is not largely overmodulated and presents minimal distortion. We noticed that the signal of PDT, that presents an intrinsic narrow EPR linewidth (0.03 mT), was distorted when reconstructed using a large modulation amplitude (0.6 mT) (Fig.S1), an observation that warrants further optimization of the algorithm. Regarding melanin detection, it could be interesting to further boost the modulation amplitude, but this would require to drastically change the configuration of the clinical EPR system due to the need to efficiently cool the modulation coils (we are using air cooling in the present configuration). Still, by increasing the modulation amplitude, we already gained a factor 2 in SNR. The cut off frequency of the low-pass filter used in the post-acquisition is another point of attention regarding the sensitivity of detection. We observed that the SNR of the melanin signal did not increase over 25 harmonics. The cutoff of the low pass filter was set by default at 700 kHz, a value that is close to 25 times the 20.4 kHz modulation frequency. We may wonder if an optimization of the cut off frequency of the low pass filter could lead to an even larger increase in SNR for melanin detection.

Despite these considerations, our comparative study definitely highlights the benefit of using multi-harmonic analysis comparatively to classical CW-EPR with a gain in sensitivity by a factor of 5. The use of a higher modulation amplitude further enhanced the sensitivity by a factor 2, leading together to an increase of sensitivity by a factor 10 (Fig. 2). As a consequence, the SNR recorded in skin melanomas was significantly improved with the ability to detect melanin in melanomas at an earlier stage of development (Fig. 3). We observed a clear relationship between the skin melanoma growth and EPR intensity. The boost in sensitivity also provided the opportunity to detect melanoma cells in deeper tissues, i.e. in iliac lymph node of mice (about 2–3 mm between the node and the coil). Thanks to the use of bioluminescence, we were able to monitor the growth in this non-superficial tissue. Again, we found a direct relationship between the EPR signal measured and the extent of the invasion. As far as we know, that is the first time that melanoma cells are detected non-invasively by EPR in deep tissues. Transposed in the clinic, this result could indicate that multi-harmonic EPR could have the capability to detect melanoma cells present more deeply under the skin, as it is the case for aggressive melanomas with high Breslow. The feasibility for studying lymph nodes in humans (that present a deeper localization) remains to be established. In lungs, we obtained mixed results due to the breath motion and longer distance between the metastases in the lungs and the coil (5–10 mm). For several mice, we were unable to record an EPR signal from the lung region. However, when the tuning was possible, we obtained a gain in SNR using multi-harmonic analysis compared to the classical CW mode. This could stimulate further research to use a triggering for synchronizing EPR recording with breath motion.

In conclusion, we demonstrate that recording the melanin signal on a clinical low frequency EPR system largely benefit from using a multi-harmonic analysis. In conditions that are close to the clinical situation, the boost in sensitivity was by a factor 10 compared to classical CW (1st harmonic) mode. Our results are paving the way for new clinical trials that will use MH clinical EPR for the characterization of pigmented skin lesions. By pushing the boundaries in the limit of detection, it is likely that other clinical applications (in oximetry and dosimetry) will also benefit of using multi-harmonic analysis.

### Supplementary Information

Below is the link to the electronic supplementary material.

Supplementary file1 (PDF 109 KB)
