# Supplementary material for: Highly Sensitive Detection of Melanin in Melanomas Using Multi-harmonic Low Frequency EPR
Source: Mol Imaging Biol. 2024 Mar 22;26(3):484–94. doi: 10.1007/s11307-024-01911-3 (PMC11211186; doi:10.1007/s11307-024-01911-3)
Supplement: Supplementary file 1 — Supplementary file1 (PDF 109 KB) [file 11307_2024_1911_MOESM1_ESM.pdf]

Highly sensitive detection of melanin in melanomas using multi-harmonic low frequency EPR

Mohammad Wehbi[1], Lionel Mignion[2], Nicolas Joudiou[2], Evelyne Harkemanne[3], Bernard Gallez[1, *]

Supplementary Fig.1.

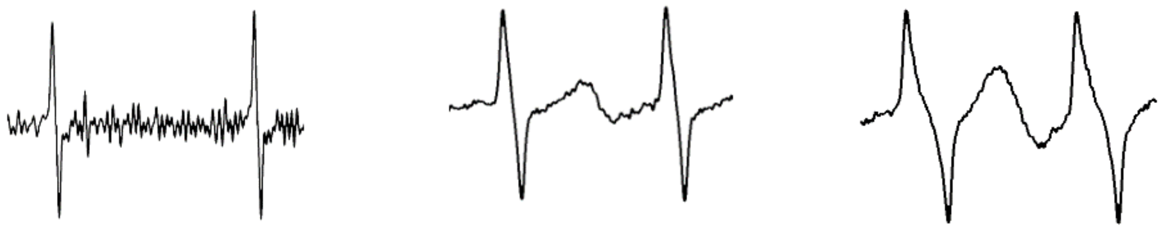

Multi harmonic analysis MA = 0.03 mT   Multi harmonic analysis MA = 0.3 mT   Multi harmonic analysis MA = 0.6 mT

Fig. S1: Multi-harmonic reconstruction of the signal recorded on a melanin phantom. The EPR spectrum contains signals coming from melanin (center) and the doublet of ^{15}N-PDT (low and high field signals) used as a reference. The analysis allows the reconstruction for a given modulation amplitude. At high modulation amplitude, the signal of melanin (that presents a large EPR linewidth, around 0.5-0.6 mT) is clearly visible without distortion while the ^{15}N-PDT appears distorted. Using a reconstruction with low modulation amplitude, the signal of melanin is almost invisible while the ^{15}N-PDT signal is not distorted with its inherent narrow linewidth (0.03 mT).
